# Relationship between perception of smile esthetics and orthodontic treatment in Spanish patients

**DOI:** 10.1371/journal.pone.0201102

**Published:** 2018-08-13

**Authors:** Belen Bolas-Colvee, Beatriz Tarazona, Vanessa Paredes-Gallardo, Santiago Arias-De Luxan

**Affiliations:** 1 Department of Orthodontics, Faculty of Dentistry, University European of Valencia, Valencia, Spain; 2 Department of Orthodontics, Faculty of Medicine and Dentistry, University of Valencia, Valencia, Spain; 3 Department of Orthodontics, Faculty of Dentistry, Cardenal Herrera University (UCH-CEU), Valencia, Spain; Kwangwoon University, REPUBLIC OF KOREA

## Abstract

One of the main objectives of orthodontic treatment is to achieve an esthetic smile. This study set out to analyze differences in the perception of smile esthetics among patients before and after receiving orthodontic treatment. 250 Spanish patients analyzed a single photograph in which, by means of computer software, midline diastema, black triangle, gingival margin of the left central incisor, and gingival (“gummy”) smile were altered. Each patient analyzed these images before and after undergoing orthodontic treatment. Patients scored the photographs on a scale from 1 to 10. Statistical analyses of each group’s level of perception were carried out, identifying significant differences in evaluations before and after treatment, and in relation to subjects’ gender and age. Patients presented significant differences in the esthetic perception of midline diastema and gummy smile anomalies after they had completed orthodontic treatment. Gender influenced the perception of smile esthetics, whereby women were significantly more critical of midline diastema, black triangle and gingival margin of the upper central incisor than men. The age variable also showed significant differences in the perception of midline diastema and black triangle anomalies. The perception of smile esthetics of some dental anomalies changes as a result of orthodontic treatment. Gender influences the perception of some of the dental anomalies studied.

## Introduction

Achieving a beautiful, esthetic smile is one of the main goals of orthodontic treatment. However, beauty has both objective and subjective dimensions. For this reason, the perception of smile esthetics depends on factors such as social and cultural awareness [1], gender [2], or the age of the observer [3]. In this context, the observer´s knowledge and experience is one of the most influential factors [1]. Several studies have compared the perception of smile esthetics among lay people with different professional backgrounds [4], general dentists [5–9], and orthodontists [10–12]. Most of these studies agree that the more specialized training the observer has received, the more sensitive his/her perception of smile esthetics will be [1]. Age is another important factor in the perception of smile esthetics, so that some irregularities such as gingival smile and black triangles are perceived differently by laypersons of different age groups [13].

Given the variations in esthetic perception and the fact that the treatment objectives of the dental professional may not coincide with the patients’ expectations, it is essential that the orthodontist is aware and understands patients’ concerns and their criteria for esthetic judgment before starting orthodontic treatment. Patients must be allowed to participate in determining treatment objectives that respond to their own perception in order to produce outcomes seen as adequate by both parties [13].

Orthodontic treatment constitutes a significant event in the life of a patient. It can be assumed that any patient who wishes to undergo orthodontic treatment is motivated by a desire to improve their smile esthetics, oral health and function, or both. Treatment may also bring about changes in dental hygiene regimes and compliance, especially among patients who receive post-procedural communication from the clinician [14]. Positive communication with the patient throughout orthodontic treatment will help to encourage and build patients’ motivation to improve their oral hygiene maintenance regime and compliance [14]. However, previous studies revealed that patients who had undergone prior treatment had no greater skill in analyzing smile esthetics [3,15].

To date, no research conducted among the general population has determined whether the perception of smile esthetics changes as a result of having received orthodontic treatment. For this reason, the objectives of this study were: 1- To compare the perception of smile aesthetics in a group of individuals before and after they had received orthodontic treatment; 2- To determine whether there are differences in perception resulting from the subjects´ gender and age.

## Materials and methods

The study protocol was approved by University of Valencia Ethics Committee for Research Involving Human Subjects, Spain (H1476712711309) and was designed following the Helsinki declaration and the STROBE statement [16]. Rights were protected by the Institutional Review Board. All subjects gave their written informed consent to take part in the study. Any data that might disclose the identity of the subjects have been omitted.

The sample consisted of 250 Spanish patients attending a private dental clinic between January 2016 and May 2017, undergoing orthodontic treatment with some kind of fixed appliance; patients were selected by Belen Bolas and Beatriz Tarazona.

The sample size was determined by a previous pilot study, which showed that a minimum of 250 subjects were necessary for an F test of an ANOVA model (MLG), with a confidence level of 95%, and considering an effect size of f = 0.15 (small-medium), to reach a power of 0.8 to detect differences between subject groups. For this sample size, the power rises to 0.99 to detect differences between a series of images showing variations of four esthetic anomalies.

### Methodology

An intraoral photograph was taken, using a Canon camera (EOS 1100D) of a patient who had just received orthodontic treatment, and presented a clinically acceptable occlusion. The image was altered digitally starting from an occlusal status considered normal. The original photograph was modified using Adobe Photoshop version CS6 Extended in order to create the most perfect mouth possible. To create a perfectly symmetrical mouth, the image was divided in two at the midline and one half was chosen and then digitally “mirrored.” Color, nose and chin were removed to eliminate possible confounding variables. Once the ideal mouth had been obtained, esthetic anomalies, with four variations each, were created using Adobe Photoshop CS6 Extended, making a total of 18 images: 16 alterations and 2 identical images of the original smile (control image) considered perfect. The four alterations were selected in reference to articles by Kokich et al. article (1999 and 2006) who conducted surveys among orthodontists with extensive clinical experience, to determine those anomalies that influence smile aesthetics decisively, and that are considered clinically common occurrences.

**Fig 1:**
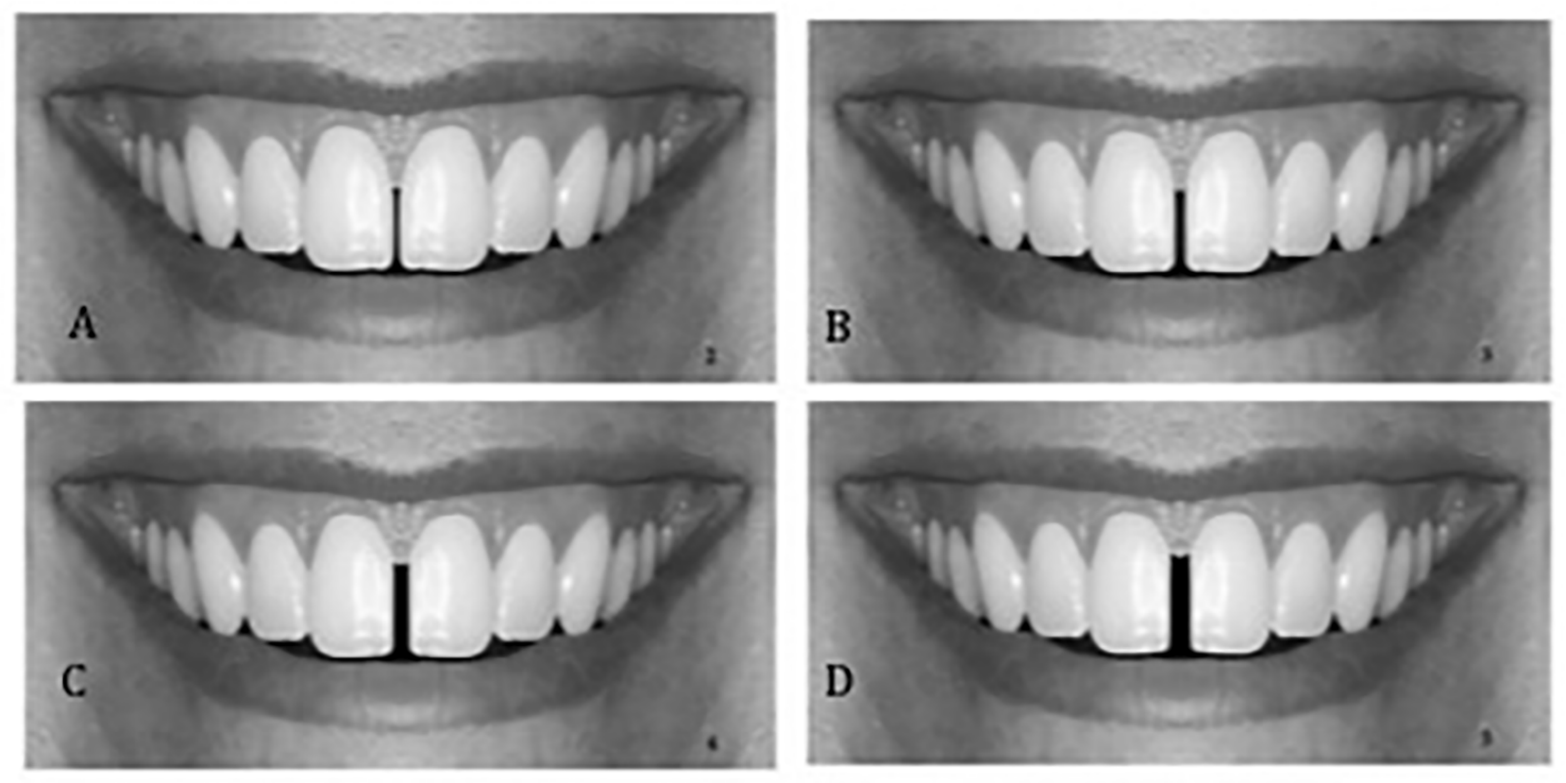
Midline diastema was created incrementally; A: 0.5mm; B: 1mm; C: 1.5mm; D: 2mm.

**Fig 2:**
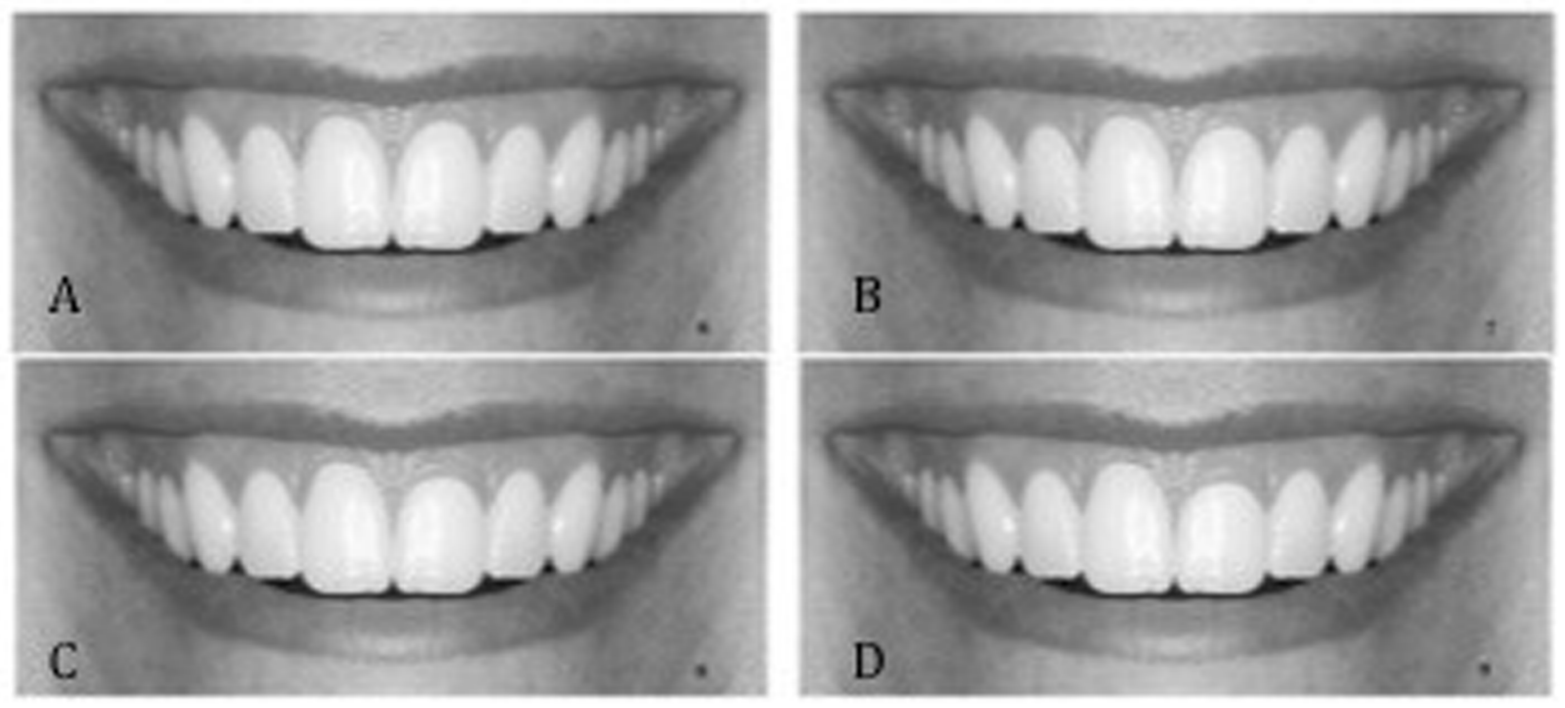
Level of gingival margin of the maxillary left central incisor was created incrementally; A, 0.5mm; B, 1mm; C, 1.5mm; D, 2mm.

**Fig 3:**
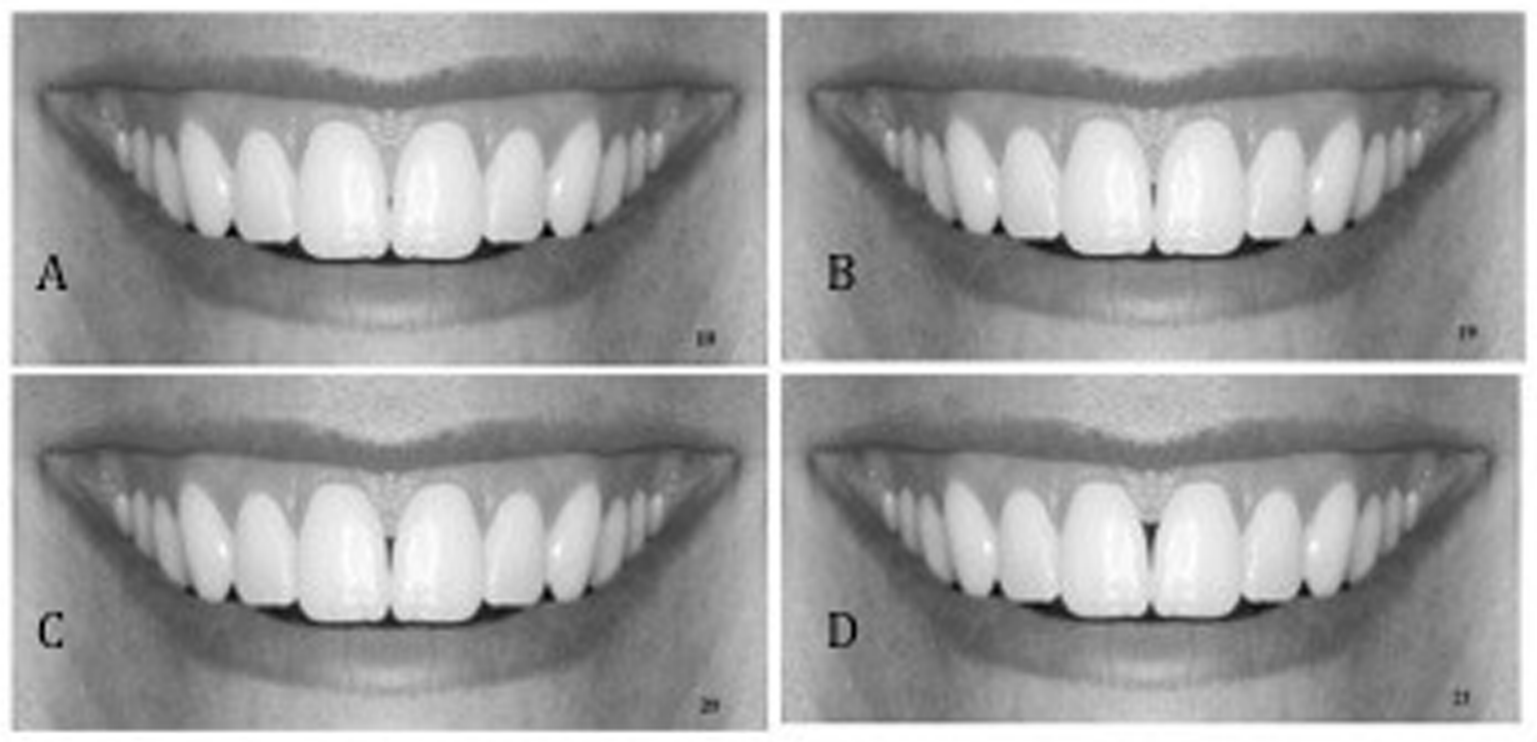
Black triangle was created incrementally; A: 1mm; B: 2mm; C: 3mm; D: 4mm.

**Fig 4:**
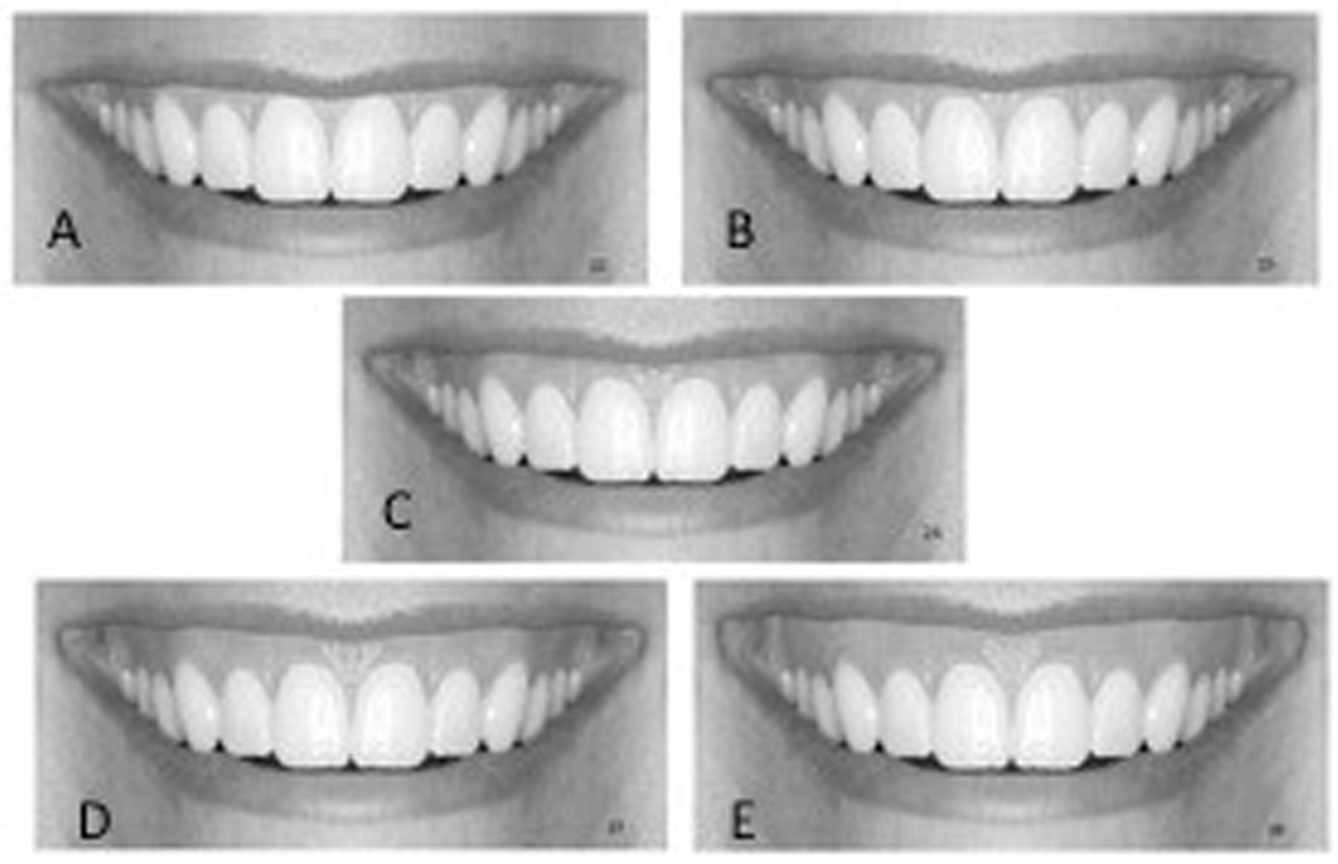
Gingiva-to-lip relationship was increased incrementally to produce a gummy smile; A, 0mm; B, 0.5mm; C, 1.5mm; D, 2mm; E, 2.5mm.

The four alterations (Figs 1–4) that were altered four times in turn were as follows:

1. Midline diastema: from 0.5 to 2mm (increments of 0.5mm).
2. Black triangle: a black triangle was created between 11 and 21, which progressively increased 1mm in apical direction in increments of 1mm (from 1 to 4 mm).
3. Gingival margin of the upper left central incisor (21): increments of 0.5mm from 0.5 mm to 2mm with respect to the upper right central incisor.
4. Gingival (“gummy”) smile: the upper lip was displaced apically in increments of 0.5mm. The first variation was made so that the lip remained at the same height as the gingival margins of the central incisors and subsequently the amount of gum shown was increased. The control image constituted one of the intermediate stages of this alteration, so that the gingival smile had four variations in addition to the control image.

All patients filled out a questionnaire on their first visit before they had been made aware of their diagnosis and treatment. The questionnaire was structured in two parts; 1. Personal data (age, sex, educational level, and reason for consultation). 2. Patients were asked to score the attractiveness of each image separately using a visual analog scale (VAS) graded from 0 (unattractive) to 10 (attractive). The sequence in which the images were shown was random, decided using the Random function of Excel 2010, whereby each image was assigned a random number between 0 and 18; the images were sorted according to these random numbers and appeared in the questionnaire accordingly. There were 5 images per page with a total of 18 images (16 alterations and two control images).

### Data analysis

To determine the reliability or reproducibility of the method, a subgroup of 50 participants repeated the first evaluation process a second time, 15 days after the first assessment. A descriptive analysis of the evaluations was made according to group, sex and age. To compare the assessments at the two different sessions, Dahlberg’s d, t-test, coefficient of variation (CV) and intraclass correlation (ICC) were applied. In order to determine whether there were differences in the evaluations according to the magnitude of the alteration and observer group affiliation, an ANOVA model of repeated measures was used, and also an ANOVA model of repeated measures with sex and age variables to assess the influence of the evaluator´s individual profile. A Brunner-Langer nonparametric model was undertaken to determine if there were differences in the impact produced by the alteration of the original image according to group. The same process was repeated for gender and age. Statistical analysis was carried out by Santiago Arias and Vanessa Paredes-Gallardo.

## Results

The final study sample consisted of 250 subjects: 166 women (65.6%) and 87 men (34.4%), with a mean age of 29.8 ± 11.7 years. Patients were divided into three age groups: 15–25 years old, 26–39 years old, and 40 years old or over.

The mean treatment time of the patients was 16.9 months (12.3–24.1).

### Reproducibility

Table 1 shows the difference in scores between the 1st and 2nd evaluations: mean, standard deviation, 95% CI, t-test for dependent samples (p-value), Dahlberg’s d, coefficient of variation (CV) and coefficient of intra-class correlation (ICC). The results have been divided into the four anomalies studied.

**Table 1:** Difference in scores between1st-2nd session: Mean, standard deviation (DE), 95% CI, t-test for dependent samples (p-value). d of Dahlberg. coefficient of variation (CV) and intraclass correlation coefficient (ICC).

	Difference of scores 1st-2nd session		CI 95%		p-value	d Dahlberg	CV (%)	ICC
	Mean	DE	Lower limit	Upper limit				
**photo 1 (ref.)**	-0.31	1.79	-0.94	0.33	0.333	1.25	12.47	0.65
**diastema 0.5mm**	0.30	1.34	-0.19	0.79	0.217	0.96	9.64	0.69
**diastema 1mm**	-0.17	0.94	-0.50	0.17	0.314	0.65	6.55	0.84
**diastema 1.5mm**	-0.16	0.83	-0.45	0.14	0.292	0.58	5.82	0.74
**diastema 2mm**	0.06	0.64	-0.16	0.29	0.570	0.44	4.42	0.67
**gingival m.21 0.5mm**	-0.07	1.45	-0.58	0.45	0.793	1.00	9.96	0.75
**gingival m.21 1mm**	-0.17	2.02	-0.88	-0.55	0.642	1.39	13.94	0.67
**gingival m.21 1.5mm**	-0.11	0.97	-0.56	0.33	0.611	0.87	8.66	0.86
**gingival m.21 2mm**	-0.24	1.07	-0.62	0.14	0.213	0.75	7.53	0.89
**black triangle 2 mm**	-0.21	1.78	-0.86	0.44	0.524	1.27	12.71	0.62
**black triangle 1 mm**	0.02	1.98	-0.69	0.72	0.965	1.36	13.65	0.67
**black triangle 3 mm**	-0.19	2.00	-0.91	0.53	0.591	1.39	13.95	0.70
**black triangle 4 mm**	0.03	1.28	-0.43	0.50	0.884	0.90	9.01	0.67
**Gingival smile 0.5mm**	0.11	1.93	-0.57	0.79	0.748	1.32	13.23	0.65
**Gingival smile 1mm**	-0.26	1.61	-0.83	0.31	0.368	1.12	11.18	0.72
**photo 24 (ref.)**	0.12	1.54	-0.43	0.66	0.659	1.06	10.62	0.76
**Gingival smile 1.5mm**	-0.29	1.63	-0.87	0.29	0.319	1.14	11.36	0.83
**Gingival smile 2mm**	-0.39	1.75	-1.01	0.23	0.207	1.23	12.32	0.86

### Midline diastema

**Fig 5:**
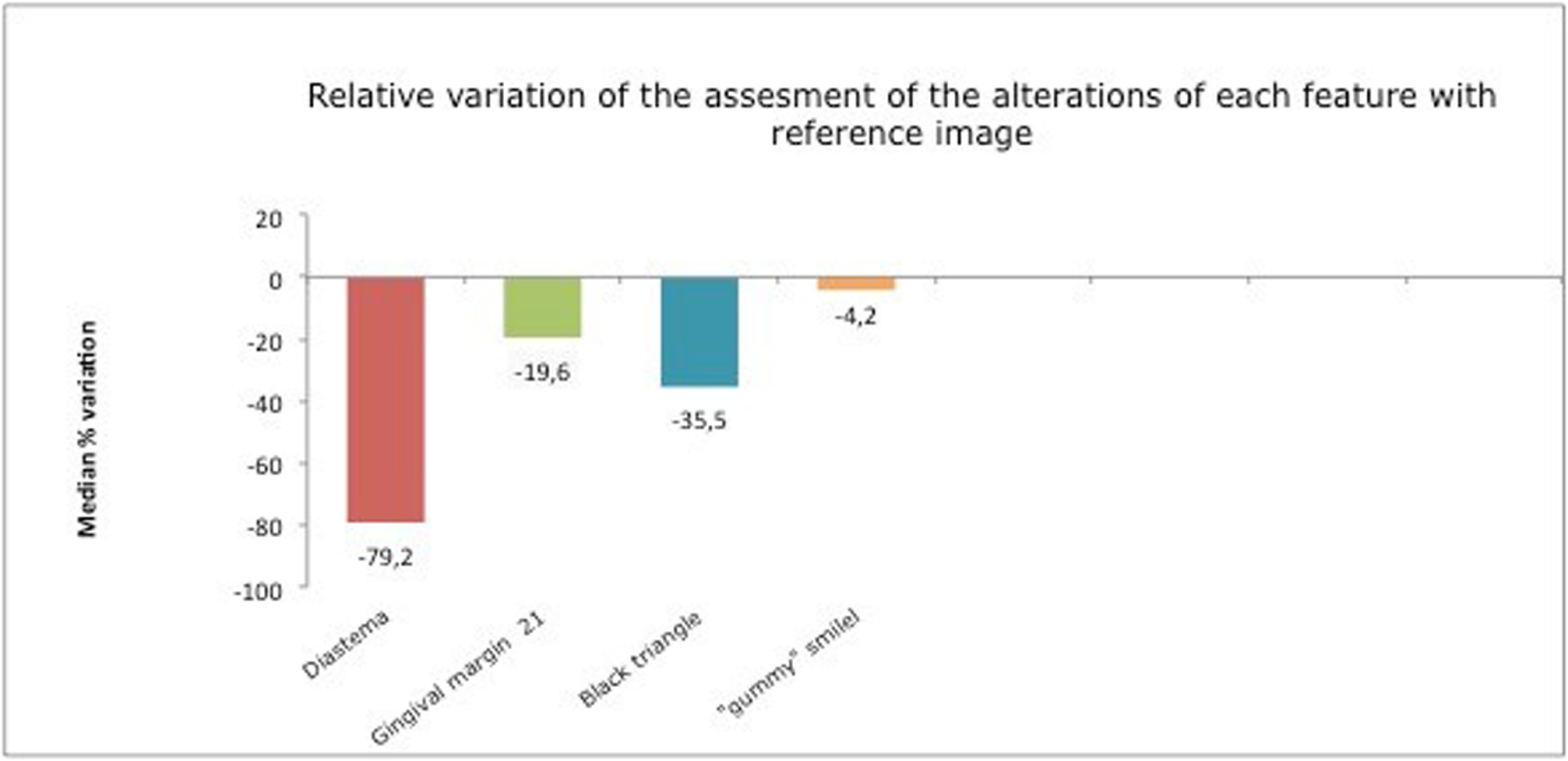
Relative variation of the assessment of the alterations of each anomaly respect to the reference image.

Fig 5 shows how midline diastema was the least esthetically acceptable anomaly. The alteration involved a (median) reduction of 79.2% in esthetic acceptability in comparison with the control image.

The diastema showed the lowest tolerance threshold among the four anomalies evaluated. Table 2 shows how patients awarded lower scores to this anomaly after treatment than before, which suggests that they found diastemas more acceptable before treatment than after.

**Table 2:** Descriptive statistics (Mean and standard deviation [SD]) by photograph. Scores for each anomaly before and after orthodontic treatment.

		Total	Pre-treatment	Post-treatment
**f2: diastema 0.5 mm**	**Mean**	2.6	2.8	2.2
	**SD**	2	1.9	1.9
**f3: diastema 1 mm**	**Mean**	2	2.4	1.6
	**SD**	1.9	2.3	1.4
**f4: diastema 1.5 mm**	**Mean**	1.2	1.5	0.9
	**SD**	1.4	1.6	1.2
**f5: diastema 2 mm**	**Mean**	0.7	0.9	0.6
	**SD**	1.1	1.3	0.8
**f6: gingival m.21 0.5 mm**	**Mean**	7.5	7.4	7.7
	**SD**	1.8	1.7	1.8
**f7: gingival m.21 1 mm**	**Mean**	6.3	6.3	6.3
	**SD**	2.2	2.1	2.3
**f8: gingival m.21 1.5 mm**	**Mean**	4.4	4.5	4.5
	**SD**	2.4	2.3	2.5
**f9: gingival m.21 2 mm**	**Mean**	3.5	3.6	3.6
	**SD**	2.2	2.2	2.3
**f18: black triangle1 mm**	**Mean**	6.5	6.6	6.4
	**SD**	2.1	2	2.2
**f19: black triangle2 mm**	**Mean**	4.6	4.4	4.8
	**SD**	2.4	2.5	2.4
**f20: black triangle3 mm**	**Mean**	4.1	4.3	3.9
	**SD**	2.4	2.4	2.4
**f21: black triangle4 mm**	**Mean**	2.5	2.8	2.3
	**SD**	1.9	2	1.8
**f22: Gingival smile 0.5 mm**	**Mean**	7.5	7.3	8
	**SD**	2.1	2	1.9
**f23: Gingival smile1 mm**	**Mean**	7.3	7.1	7.6
	**SD**	1.9	1.8	1.9
**f25: Gingival smile1.5 mm**	**Mean**	6.1	5.9	6.3
	**SD**	2.4	2.3	2.2
**f26: Gingival smile2 mm**	**Mean**	6	5.4	6.4
	**SD**	2.6	2.5	2.4

**Fig 6:**
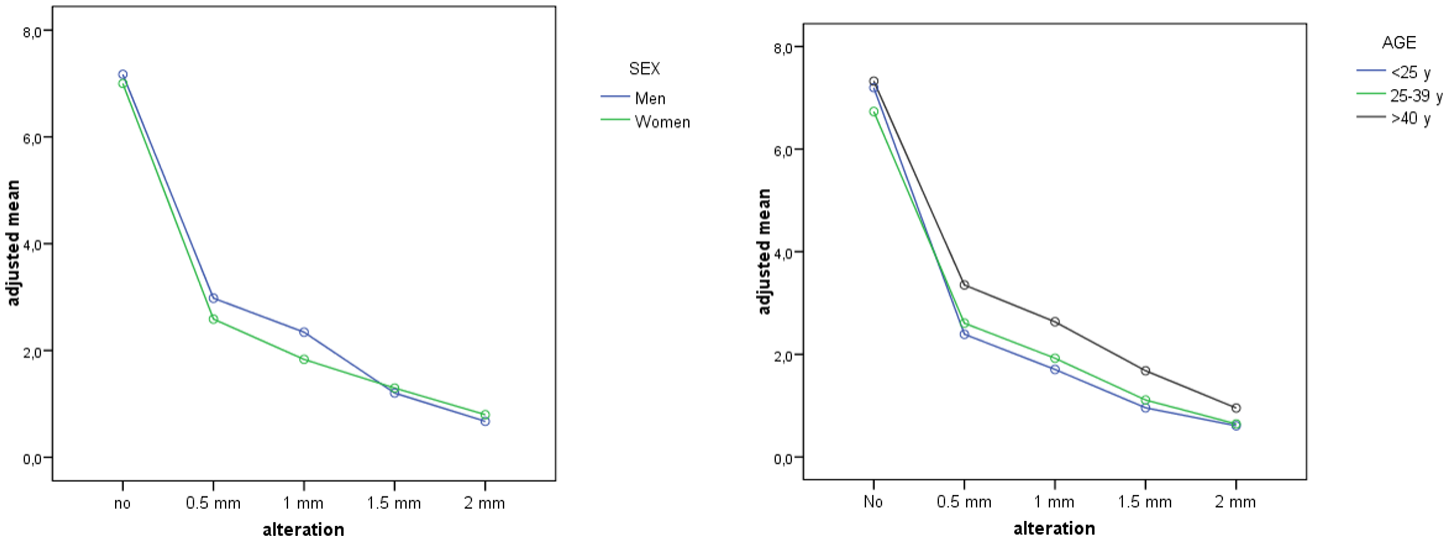
Estimated n measures for each alteration of the midline diastema anomaly for sex (6a) and age (6b).

Statistically significant differences were found between genders (Fig 6a), whereby women were more critical of diastema than men. As for the age variable, the diastema anomaly was more acceptable to older than younger participants. For example, for a 0.5 mm midline diastema, older subjects awarded the image a significantly higher score than younger subjects (p = 0.028); the same difference was seen for the 1 mm (p = 0.022) and the 1.5 mm diastemas (p = 0.018). For the maximum alteration of 2mm, there were no significant differences between age groups (Fig 6b).

### Black triangle

This anomaly received the second lowest scores. The first and slightest alteration of the black triangle caused a drop in the score of 35.5% (Fig 5). Table 2 shows that there were no differences in patients’ evaluations of any of the black triangle variations before and after orthodontic treatment.

**Fig 7:**
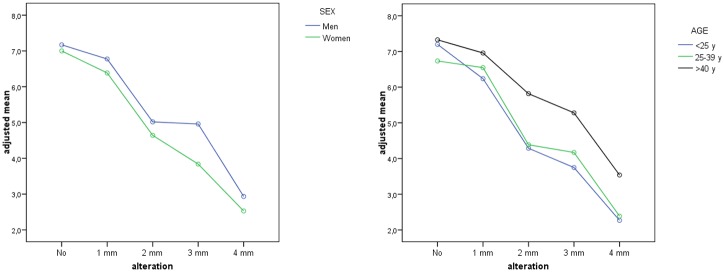
Estimated measures for each alteration of the black triangle anomaly for sex (7a) and age (7b).

No statistically significant differences were found between genders, and both sexes valued the slighter black triangle variations equally (Fig 7a); but for 3 mm (p<0.001) and 4 mm (p = 0.096) black triangles, men were found to be more tolerant than women. For the 2 mm variation, subjects aged under 40 were significantly more critical of the images than older subjects (Fig 7b).

### “Gummy” smile

Gummy smile was found to be the most acceptable anomaly, with a reduction in scores of only 4.5% in comparison with the control image (Fig 5). As shown in Table 2, patients who had completed their orthodontic treatment awarded higher scores for gummy smile variations of 0.5 mm and 0.2mm than those who had not started treatment. So, subjetcs who had ended their orthodontic treatment preferred the gingival smile.

No statistically significant differences were found between gender and age groups.

### Gingival margin of the left central incisor

Alterations of the gingival margin of tooth 21 led to a reduction in scores of 19.6% compared with the control image (Fig 5). As shown in Table 2, no statistically significant differences were found between scores before and after treatment for any of the images of the gingival margin anomaly.

**Fig 8:**
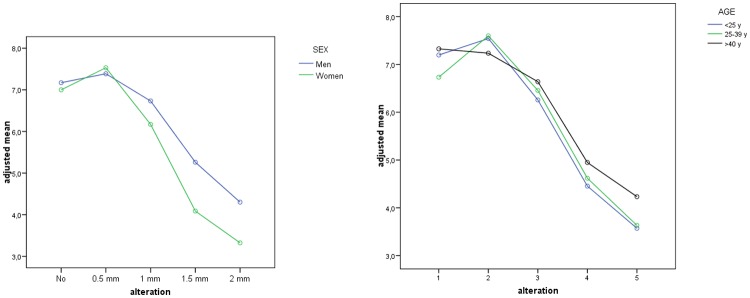
Estimated measures for each alteration of the gingival margin anomaly for sex.

Men found moderate alterations of the gingival margin more acceptable than women. For example, for 1 mm of alteration, men awarded a higher score than women (p = 0.063). Differences grew as the degree of the anomaly increased (p <0.001) (Fig 8). But no statistically significant differences were found between groups.

## Discussion

The aim of this study was to compare the perception of smile esthetics among group of Spanish patients before and after receiving orthodontic treatment, analyzing whether there were differences between the genders and between age groups. No other study in the literature has made such an analysis.

Intra- and inter-observer error was low, so reproducibility was high; the coefficient of variation (CV) and the Dahlberg´s d-value were very low. We cannot compare this result with any other study, as no other has calculated reproducibility or compared changes in perception of smile esthetics resulting from subjects having received orthodontic treatment.

The four anomalies used to analyze perceptions of smile esthetics were selected by consulting orthodontists and general dentists on the basis of their frequent occurrence and clinical significance, as in the study by Kokich et al. (2009) [10].

Analyzing the results for each of the four anomalies assessed, evaluation of the midline diastema fell by 79.2%, compared with the control image, showing how the presence of a midline diastema is a decisive factor affecting esthetic perceptions negatively, as shown by Rosenstiel and Rashid [17]. Moreover, statistically significant differences were found between the two evaluations before and after treatment. These results differ from an earlier study, which did not find these differences [15]. They could be due, firstly, to the fact that the former study was carried out among dental students, unlike the present work which drew subjects from the general population, and secondly, because the average age of the present sample was older.

In subjects´ perception of black triangle, the score decreased by 35% when the anomaly was present. But no statistically significant differences were found between evaluations made before and after treatment. No previous article has compared the perception of black triangles in terms of whether or not subjects have received orthodontic treatment, but those that have compared perception between different populations affirm that triangles larger than 3 mm are detected by the general population [10].

Gingival, “gummy” smile was found to be the most acceptable anomaly, although significant differences were found between evaluations made before and after treatment. According to the literature on evaluations of factors influencing smile aesthetics, this anomaly presents widely varying results. Some authors believe that smile esthetics are compromised by a gingival smile of 1mm [18], 2mm [19,20], 2.5mm [21] or up to 3mm [22]. However, España et al. [15] did not find statistically significant differences among their sample of dentistry students at different stages of the degree course, who evaluated gingival smiles of 1, 2, or 3 mm. These differences in results might be explained by the fact that different works investigated different sample groups, and the gingival smiles evaluated were not all of the same magnitude.

No significant differences were found when alterations to the gingival margin were analyzed. These results agree with An et al. [23] and España et al. [15], although the earlier studies used different evaluation methods and analyzed the upper right central incisor, rather than the left as in the present study.

Analyzing the overall results, the present study found statistically significant differences between genders in the perception of midline diastema, black triangle and gingival margin, whereby women were more critical of these anomalies than men. These results agree with those of Abu Alhaija et al.[12] who also analyzed midline diastema (and other anomalies), and with other studies that have compared midline deviation [24] or smile esthetics in different malocclusions [18,25]. But results obtained by other authors have shown that gender does not influence the perception of smile esthetics [4,6].

Similarly, statistically significant differences were found in the perceptions of two of the anomalies evaluated between the three age groups. For the esthetic perception of midline diastema, the present results agree with Rosenstiel and Rashid [17], although the latter authors used a different method, and with Rodrigues et al.[26], who also found differences between age groups, young people being more critical. As for black triangle, statistically significant differences were found, whereby patients aged over 40 years were found to be less critical. These data agree with Pithon et al. [27], who observed that esthetic perception became less critical with age.

The fact that the present study used an image of a woman’s smile may constitute a limitation, as authors such as Anderson et al. [28] have found that the perception of smile esthetic are influenced by whether the image is of a man or a woman.

## Conclusions

Perceptions of midline diastema and gummy smile alter significantly between evaluations made by patients before and after orthodontic treatment. Gender influences the perception of the smile esthetics whereby women are more critical of midline diastema, black triangle and the gingival margin of the upper central incisor than men. Age also influences the esthetic perception of midline diastema and black triangle, whereby younger age groups are more critical than older subjects.
